# Dual-Task Interference on Early and Late Stages of Facial Emotion Detection Is Revealed by Human Electrophysiology

**DOI:** 10.3389/fnhum.2019.00391

**Published:** 2019-11-08

**Authors:** Amélie Roberge, Justin Duncan, Daniel Fiset, Benoit Brisson

**Affiliations:** ^1^Département de Psychologie, Université du Québec à Trois-Rivières, Trois-Rivières, QC, Canada; ^2^Département de Psychoéducation et de Psychologie, Université du Québec en Outaouais, Gatineau, QC, Canada; ^3^Département de Psychologie, Université du Québec à Montréal, Montreal, QC, Canada

**Keywords:** facial expression, emotion, dual-task interference, psychological refractory period, central attention

## Abstract

Rapid and accurate processing of potential social threats is paramount to social thriving, and provides a clear evolutionary advantage. Though automatic processing of facial expressions has been assumed for some time, some researchers now question the extent to which this is the case. Here, we provide electrophysiological data from a psychological refractory period (PRP) dual-task paradigm in which participants had to decide whether a target face exhibited a neutral or fearful expression, as overlap with a concurrent auditory tone categorization task was experimentally manipulated. Specifically, we focused on four event-related potentials (ERP) linked to emotional face processing, covering distinct processing stages and topography: the early posterior negativity (EPN), early frontal positivity (EFP), late positive potential (LPP), and also the face-sensitive N170. As expected, there was an emotion modulation of each ERP. Most importantly, there was a significant attenuation of this emotional response proportional to the degree of task overlap for each component, except the N170. In fact, when the central overlap was greatest, this emotion-specific amplitude was statistically null for the EFP and LPP, and only marginally different from zero for the EPN. N170 emotion modulation was, on the other hand, unaffected by central overlap. Thus, our results show that emotion-specific ERPs for three out of four processing stages—i.e., perceptual encoding (EPN), emotion detection (EFP), or content evaluation (LPP)—are attenuated and even eliminated by central resource scarcity. Models assuming automatic processing should be revised to account for these results.

## Introduction

Facial expressions of emotions are a powerful non-verbal social tool for externalizing internal states and making these salient to other individuals. As such, rapid and effortless (i.e., automatic) processing of potential social threats—even ones that lie outside of attention—would provide a clear evolutionary advantage. Although this view has prevailed for some time (e.g., Palermo and Rhodes, 2007), several researchers now question the extent to which the processing of facial emotions is automatic.

Most studies that have investigated the relationship between attention and the processing of emotional facial expressions have relied on visual-spatial attention paradigms to manipulate the focus of attention toward specific items or locations in the visual field, or toward specific stimulus features. In visual search paradigms, for instance, participants are required to orient attention toward pre-defined targets (i.e., task-relevant items) that are presented among distractors (i.e., task-irrelevant items; for review, see Carretié, 2014). Using a variant of this paradigm, Vuilleumier et al. (2001) found evidence of increased left amygdala activity in response to fearful (vs. neutral) facial expressions presented at both attended and unattended locations, consistent with the automatic processing account.

However, using a more difficult main task, Pessoa et al. (2002) failed to obtain the same increase in amygdala activity when emotional faces were task-irrelevant (for similar results, see also Pessoa et al., 2005; Bishop et al., 2007; Mitchell et al., 2007; Alpers et al., 2009; Kellermann et al., 2011; Sebastian et al., 2017). The authors proposed that processing of irrelevant emotional faces occurs only when the main task does not succeed at entirely monopolizing attentional resources, and leaves spare resources that can be deployed toward task-irrelevant stimuli (as per Lavie, 1995). This line of thought implies that emotional face processing requires visual-spatial attention. Indeed, if the main task is difficult enough, it will monopolize all resources, leaving none to be deployed toward irrelevant—even highly salient—emotional faces, which will in turn not be processed during the execution of the main task.

Interestingly, the results of Pessoa et al. (2002) imply that central attention resources might be a mediator in this equation. Central attention is used in the preferential allocation of processing resources toward a specific task (Pashler, 1991), and appears to operate at stages coinciding with short-term memory consolidation (Johnston et al., 1995), or response selection (for review, see Lien and Proctor, 2002). One well-suited method for studying the effects of central attention is the psychological refractory period (PRP) dual-task paradigm (Telford, 1931; Pashler, 1984). The PRP consists in experimentally manipulating the degree to which processing of two tasks overlaps, with each task requiring a speeded and accurate response from participants: Often a prioritized first task target (T_1_), and a non-prioritized second task target (T_2_). By shortening and lengthening the delay between T_1_ and T_2_ onsets (i.e., stimulus onset asynchrony, SOA), processing overlap is increased and decreased, respectively. In the absence of stimulus masking, greater central overlap (i.e., shorter SOA) typically leads to slower response times (RT) to T_2_, compared to when there is little or no overlap (i.e., longer SOA). This lengthening of RT to T_2_ as SOA decreases (the so-called PRP effect) is explained by the fact that central operations (e.g., response selection) cannot be carried simultaneously on two concurrent tasks (Pashler, 1994; Tombu and Jolicoeur, 2002).

To investigate the impact of central attention modulation on processing of emotionally expressive faces, Tomasik et al. (2009) have employed a clever PRP paradigm consisting in the auditory discrimination between a pure tone and noise (T_1_), and the visual discrimination between ambiguous (hard) and unambiguous (easy) happy and angry faces (T_2_). The logic was that if perceptual stages of emotion processing are automatic, then the effect of T_2_ difficulty on RT should be absorbed by cognitive “slack” at short SOA (i.e., locus-of-slack). Their results indicated that concurrent central processing of T_1_ indeed interfered with perceptual processing stages of emotionally expressive faces. However, due to the use of ambiguous stimuli, this paradigm made it difficult to dissociate perceptual from decisional effects.

Though neuroimaging tools such as fMRI have allowed crucial advances in understanding and mapping various processes of the mind, such methods lack the necessary temporal resolution to capture often subtle and transient impacts attention modulation may have on emotional face processing (Eimer et al., 2003). Electrophysiology, on the other hand, is ideally suited for this purpose, and Shaw et al. (2011) have integrated such measures to their paradigm in order to isolate the stages affected by PRP. Specifically, they investigated the N2pc, an event-related potential (ERP) index of visual-spatial attention (Luck and Hillyard, 1994; Eimer, 1996; Brisson et al., 2007). While their behavioral measures reflected a PRP effect at short SOA, N2pc amplitude was similar across all SOA conditions, indicating that emotion perception is carried automatically, before the central bottleneck. However, the N2pc is at best an indirect measure of facial emotion processing, and it is also likely that the N2pc at short SOA was observed because the auditory task was too easy (Brisson and Jolicoeur, 2007a,b,c; Lien et al., 2011).

Several ERPs have directly been associated with emotional face processing (for review, see Eimer and Holmes, 2007; Vuilleumier and Pourtois, 2007). The first component of interest for our purpose is the early posterior negativity (EPN). The EPN emerges as a negative amplitude at occipital electrodes between approximately 200 ms and 300 ms post-stimulus, when activity elicited by neutral stimuli is subtracted from emotional stimuli. It is thought to reflect increased visual processing of emotional content (for review, see Hajcak et al., 2011), and has been observed for several categories, including faces (e.g., Sato et al., 2001; Schupp et al., 2004; Mavratzakis et al., 2016). Interestingly, the EPN can be observed when facial expressions are task-irrelevant, suggesting that the visual processes it indexes are not dependent upon visual spatial attention (Rellecke et al., 2011).

The second and third components are the early frontal positivity (EFP) and late positive potential (LPP), which both emerge as a relative positive amplitude increase in response to emotional—compared to neutral—faces (e.g., Eimer and Holmes, 2007; Holmes et al., 2009). The EFP, onsets as early as 110–130 ms post-stimulus onset at frontocentral electrodes, and it is thought to reflect an early prefrontal processing stage such as rapid detection of emotional content (Eimer and Holmes, 2007). The LPP typically onsets around 300 ms post-stimulus at centro-parietal (Hajcak et al., 2011) or frontocentral electrodes (Holmes et al., 2009), and it can persist well beyond 1,000 ms post-stimulus (e.g., Cuthbert et al., 2000; Hajcak and Olvet, 2008). It is thought to reflect later and higher-level processing stages, such as sustained and elaborate analysis of emotional content, self-reported arousal, and motivational salience of stimulus content (Weinberg and Hajcak, 2010). Visual-spatial attention appears to be necessary for the emotion processing indexed by the EFP and LPP, both for task-irrelevant emotional faces presented in peripheral and central vision; although the EFP seems somewhat more resilient when faces are presented centrally (Eimer et al., 2003; Holmes et al., 2003, 2006).

An interesting study combining all three above ERP components looked at the impact of cognitive load on the processing of centrally-presented expressive faces in low and high trait anxiety individuals using an *n*-back paradigm (Holmes et al., 2009). Whereas the EPN of both groups was unaffected by the increase in cognitive load, the EFP and LPP amplitudes of low (but not high) trait anxiety individuals were significantly reduced. These results suggest that early perceptual processing of emotional stimuli is impervious to cognitive load, whereas early and late frontal processing are susceptible. However, the task was administered in a block-design, conflating the antagonistic effects of central processing and task preparation.

The fourth and final electrophysiological component we isolated for our study is the face-sensitive N170. The N170 is typically observed at occipito-temporal sites 150–200 ms post-stimulus, and is often found to exhibit greater amplitudes for faces, compared to other object categories (Bentin et al., 1996). It is usually taken to reflect the structural encoding of faces (Eimer, 2011), though it has also been likened to an eye processor, more specifically (e.g., Rousselet et al., 2014). Though earlier studies found no clear evidence of emotional modulation of this ERP (e.g., Eimer et al., 2003), a more recent meta-analysis found that on the whole, research supports the hypothesis of emotional modulation (Hinojosa et al., 2015) across various stimulus parameters (Schindler et al., 2019), and this appears to be most reliable for fearful facial expressions (Turano et al., 2017). Of interest to our current research investigation, the N170 also appears resistant to perceptual load (Müller-Bardorff et al., 2016), suggesting that it might also be resilient to central resource scarcity. Thus, we opted to include the N170 as a final emotion processing electrophysiological marker.

The goal of the present study was to re-examine the question of whether processing of facial emotions requires central attention using ERP components that have been directly linked to emotion processing. We measured EPN, EFP, LPP, and N170 emotion responses during a PRP paradigm combining a difficult auditory task (Brisson and Jolicoeur, 2007a,b,c), a variable SOA, and an emotion detection task on faces presented in central vision. Although automatic early perceptual processing of emotional faces has been reported for the visually-evoked P1 (e.g., Holmes et al., 2009), we chose to overlook this ERP because of the high likelihood that this effect is, in fact, an artifact of low-level image properties (Bekhtereva et al., 2015). If the stages of emotional face processing indexed by the EPN, EFP, LPP, and N170 evoked potentials require central attention, then the emotional modulation of their amplitude should be reduced or eliminated at shorter compared to longer SOAs; otherwise, it should remain similar across all SOA durations.

## Materials and Methods

### Participants

Thirty-two participants completed the experiment for financial compensation. Five participants were excluded from the analyses (three for numerous artifact rejection or technical problems and two for low accuracy or excessively slow responses; see “EEG Recording and Analysis” and “Results” sections below), such that our final sample comprised 27 participants (19 women) between the ages of 19 and 30 years (*M* = 22.29 years, *SD* = 2.81). Based on self-report, all participants were neurologically intact, had normal hearing, and had normal or correct-to-normal visual acuity. Written consent was obtained from each participant prior to beginning the experiment. The procedure was vetted by the research ethics committee at the Université du Québec à Trois-Rivières.

### Stimuli and Apparatus

The experiment was programmed in E-prime 2.0 and stimuli were presented on a 16-inch CRT computer monitor (1,024 × 768 resolution, 60 Hz refresh rate). A black background was used during the whole experiment. Participants sat approximately 67 cm from the monitor. Stereo tones (T_1_) were emitted by two loudspeakers placed on either side of the monitor, and faces (T_2_) were presented centrally on the visual display. T_1_ could be one of four pure frequency tones (200, 430, 926 or 2,000 Hz). T_2_ was one of forty face images (10 male and 10 female; neutral and fearful expressions) taken from the Radboud Face Database (Langner et al., 2010). Faces were converted to grayscale and aligned on the positions of the main internal features (i.e., eyes, nose, and mouth) using translation, rotation and scaling. A gray oval background spanning 4 degrees horizontally was also added to hide external facial features (face contour, ears, and hair). Finally, image luminance histograms and spatial frequency spectra were equated between stimuli with the SHINE toolbox (Willenbockel et al., 2010) for Matlab (Natick, MA, USA). This step is crucial, because low-level image properties are known to exert influence on early visual processes independently from semantic content, such as those indexed by the P1 visually-evoked potential (Rossion and Caharel, 2011), and even on higher level face-sensitive regions, such as the fusiform face area (Weibert et al., 2018), which is largely believed to be the cortical source of the N170 (Sadeh et al., 2010; but see however Jacques et al., 2019).

### Procedure

After initially hearing each of the four possible tones (in ascending order of frequency) and seeing a single face for each expression condition (i.e., fearful or neutral), participants performed a practice block of 72 trials. These were divided into 48 auditory (T_1_) single-task trials, 12 visual (T_2_) single-task trials, and 12 auditory-visual (i.e., T_1_ + T_2_) dual-task trials. This practice block was followed by six experimental blocks comprising 160 trials each (totaling 960 trials).

**Figure 1:**
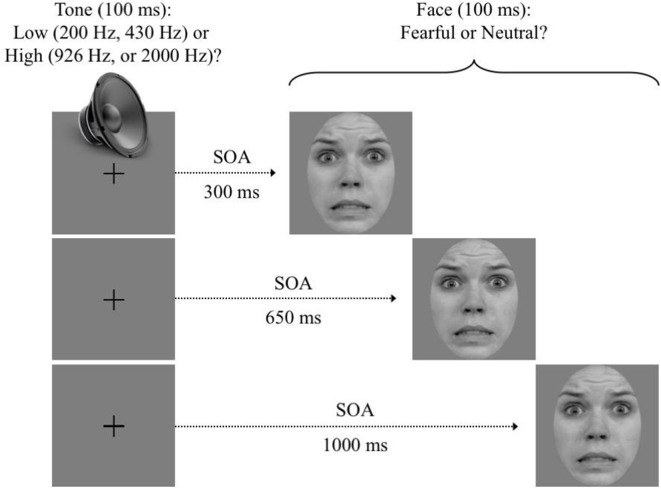
Trial sequence. After participants self-initiated the trial, a pure tone was emitted for 100 ms. Then, following a variable stimulus onset asynchrony (SOA) of 350, 650, or 1,000 ms, a face was displayed for 100 ms. Instructions emphasized a quick and accurate answer to both targets. Note: use of RaFD images in figures is permitted.

Each dual-task trial sequence (see Figure 1) was self-initiated with a simultaneous press on the “S” and “J” keys with the left and right index fingers, respectively. This instigated the presentation of a central fixation point at the center of the screen. After a variable delay of 300–500 ms, an auditory tone (T_1_) was emitted (100 ms). Then, following a variable SOA (300, 650, or 1,000 ms), a face (T_1_) was presented centrally (100 ms), replacing the fixation point. All possible T_1_, SOA, and T_2_ values were randomly intermixed within each block and across trials.

Separate two-choice responses were required on each trial. The first response was to indicate if T_1_ was a low (200 or 430 Hz) or high (926 or 2,000 Hz) frequency tone. This was done by pressing the “A” (low frequency; left middle finger) and “S” (high frequency; left index) keys. The second response was to indicate if T_2_ exhibited a fearful or neutral expression. This was done by pressing the “J” (fearful; right index) and “K” (neutral, right middle finger) keys. Instructions emphasized a quick and accurate response to each target as it unfolded (i.e., prioritization of T_1_).

Five-hundred to 750 ms after the last response was registered, the central fixation point was replaced by visual feedback. Specifically, a “+” (i.e., correct) or a “−” (i.e., incorrect) symbol was presented to the left (i.e., T_1_ feedback) and right (i.e., T_2_ feedback) of the central fixation location. The feedback remained visible until the next trial was initiated. Verbal instructions emphasized that participants maintained a central eye fixation throughout the trial sequence, and blink only while the feedback appeared on the screen. Participants were also allowed periodic breaks between blocks if desired.

### EEG Recording and Analysis

The EEG was recorded from 64 active Ag/AgCl electrodes (ActiCHamp system with actiCap, Brain Products Inc.,) mounted on an elastic cap. Electrodes were placed according to the International 10/10 system, with the exception that the TP9 and TP10 electrode sites were displaced 1 cm below their respective original sites, over the mastoids. All electrodes were recorded with a left-mastoid reference, and the data were re-referenced offline to then common average reference. Two additional electrodes, placed near external canthi, were used to record horizontal (HEOG). Electrode impedance was kept below 15 kΩ. EEG data were digitized at 500 Hz and band-pass filtered (0.01–30 Hz) offline, prior to averaging.

Electrodes of interest were FC1, FCz, and FC2 for the EFP and LPP, and O1, Oz, and O2 for the EPN (Holmes et al., 2009). P7 and P8 (i.e., electrodes with largest amplitudes in the 150–200 ms time-window) were used for the N170 (see Rousselet et al., 2014). Only T_1_-correct trials with RT below 2,000 ms were included in the averaged waveforms. Trials with eye blinks (amplitude in excess of ±80 μV at Fp1) or other artifacts (i.e., amplitudes exceeding ±80 μV at electrodes of interest) were rejected. Participants with more than 50% rejected trials in at least one experimental condition (*N* = 3) were excluded from any further analysis. Of the remaining participants, an average 78.66% (*SD* = 8.25) of all 300 ms SOA trials, 80.81% (*SD* = 7.55) of all 650 ms SOA trials, and 82.74% (*SD* = 7.25) of all 1,000 ms SOA trials, remained after artifact rejection.

For each experimental condition, EEG data were averaged across trials over a window starting 100 ms prior to T_2_ onset, and ending 800 ms after T_2_ onset. The 100 ms pre-T_2_ onset period was used for baseline correction. EFP, LPP, EPN, and N170 measures were obtained by subtracting the neutral face waveforms from the fearful face waveforms. This eliminated overlap in activity between conditions, including residual Task 1 activity at shorter SOAs, and left what was unique to facial emotion processing.

The EFP was measured at FC1, FCz, and FC2 from 130 to 220 ms after T_2_ onset; and the LPP was measured at the same electrodes from 500 to 800 ms. The N170 was measured at P7 and P8 from 150 to 200 ms, and the EPN was measured at O1, Oz, and O2 from 220 to 280 ms after T_2_ onset (e.g., Mavratzakis et al., 2016).

## Analyses and Results

Only trials with accurate responses to T_1_, RT to T_1_ (RT_1_) between 100 and 2,000 ms, and RT_2_ between 100 and 5,000 ms were kept for the analyses. Participants with mean RT_1_ slower than 1,500 ms or with less than 70% correct responses were rejected (*N* = 2).

Mean percent correct responses and reaction times for both T_1_ and T_2_ were each submitted to a 3 (SOA: 300 ms, 650 ms, 1,000 ms) × 2 (T_2_ Expression: fearful, neutral) repeated measures analysis of variance (ANOVA). Electrophysiological data extracted from the Fearful–Neutral difference EFP, LPP, and EPN waveforms were each analyzed with a 3 (SOA) × 3 [Electrode: FC1, FCz, FC2 (EFP, LPP); or O1, Oz, O2 (EPN)] repeated measures ANOVA. The N170 Fearful–Neutral difference waveforms were analyzed with a 3 (SOA) × 2 (Electrode: P7, P8) repeated measures ANOVA. A Bonferroni correction was applied for all analyses.

### Behavioral Results

Average RT and accuracy to T_1_ and T_1_ are reported in Table 1 for each SOA, and valence condition.

**Table 1:** Mean reaction time (RT, in milliseconds) and accuracy (ACC, percent correct) for Task 1 and Task 2 are displayed as a function of SOA and T_2_ facial expression.

		Stimulus onset asynchrony (SOA)					
		300 ms		650 ms		1,000 ms	
		RT	ACC	RT	ACC	RT	ACC
Task 1
	Fear	859.72 (234.97)	87.63 (5.32)	920.94 (271.26)	88.44 (5.17)	1,022.36 (382.61)	89.81 (5.28)
	Neutral	859.18 (230.58)	87.70 (6.63)	934.04 (281.92)	88.81 (5.33)	1,032.63 (387.96)	89.33 (5.09)
Task 2
	Fear	937.32 (165.9)	92.48 (7.92)	763.23 (135.37)	91.04 (8.28)	690.34 (116.6)	91 (7.36)
	Neutral	967.33 (161.73)	90.78 (6.44)	789.56 (133.56)	93.11 (4.65)	710.06 (123.51)	93.19 (5.23)

*The standard error of the mean is in parentheses*.

#### Auditory Task

There was a significant effect of SOA on T_1_ accuracy, *F*_(2,52)_ = 8.34, *p* = 0.001, $\eta_{\text{p}}^{2}$ = 0.24, such that accuracy increased with lengthening SOA. There was also a main effect of SOA on RT_1_, *F*_(2,52)_ = 23.07, *p* < 0.001, $\eta_{\text{p}}^{2}$ = 0.47, which increased with lengthening SOA. Thus, there was a slight speed-accuracy trade-off, which was most likely caused by T_2_ onset at shorter SOAs precipitating the T_1_ response before processing was completed. Finally, there was a main effect of T_2_ Expression on RT_1_, *F*_(1,26)_ = 4.85, *p* = 0.04, $\eta_{\text{p}}^{2}$ = 0.16, such that RT_1_ was shorter when fearful (vs. neutral) faces were presented. No other effects reached significance, all *F*s < 1.10.

#### Visual Task

As is typically observed in PRP studies in which T_1_ is not masked, there was no effect of SOA on T_2_ accuracy, *F* < 1. The main effect of T_2_ Expression on T_2_ accuracy was also non-significant, *F* < 1. However, there was a significant SOA X T_2_ Expression interaction effect on T_2_ accuracy, *F*_(2,52)_ = 10.37, *p* < 0.001, $\eta_{\text{p}}^{2}$ = 0.29. This interaction was qualified by a significant T_2_ Expression effect for the longer 1,000 ms SOA, *t*_(26)_ = −2.30, *p* = 0.03, *d* = 0.40, but not the shorter 300 ms (*t*_(26)_ = 1.18, *p* = 0.25, *d* = 0.25) or 650 ms (*t*_(26)_ = −1.64, *p* = 0.113, *d* = 0.29) conditions. Importantly, the expected PRP effect of SOA on RT_2_ was observed, *F*_(2,52)_ = 265.06, *p* < 0.001, $\eta_{\text{p}}^{2}$ = 0.91, as RT were lengthened with SOA shortening. A main effect of T_2_ Expression on RT_2_ was also observed, *F*_(1,26)_ = 15.98, *p* < 0.001, $\eta_{\text{p}}^{2}$ = 0.38, with faster RT recorded for fearful (vs. neutral) faces. The SOA X T_2_ Expression interaction was nonsignificant, *F* < 1.

### Electrophysiological Results

**Figure 2:**
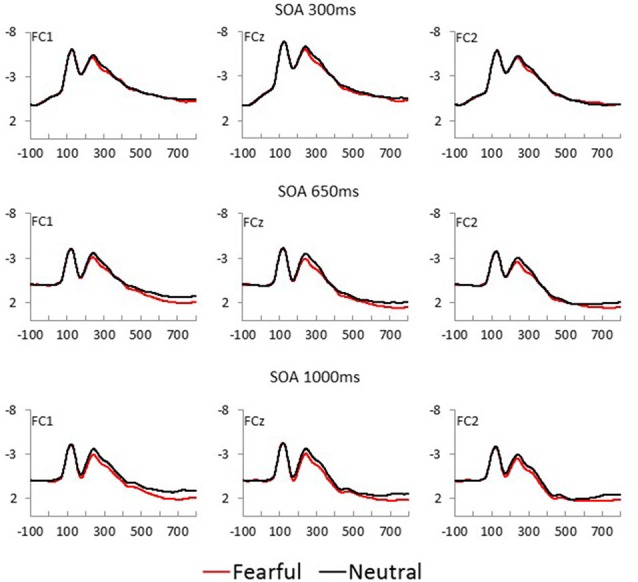
Fearful (red) and Neutral (black) waveforms at frontocentral sites are displayed as a function of electrode position, for each SOA condition (top = 300 ms, middle = 650 ms, and bottom = 1,000 ms).

**Figure 3:**
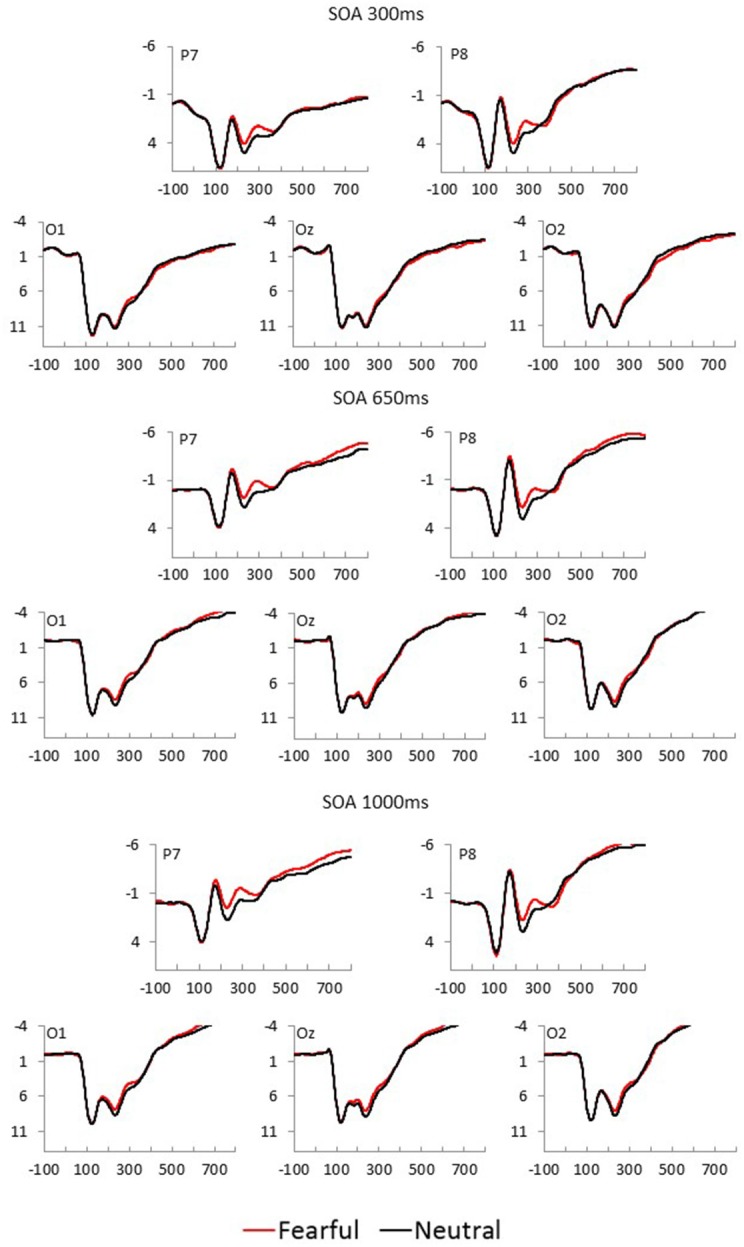
Fearful (red) and Neutral (black) waveforms at parietal and occipital sites are displayed as a function of electrode position, for each SOA condition (top = 300 ms, middle = 650 ms, and bottom = 1,000 ms).

**Figure 4:**
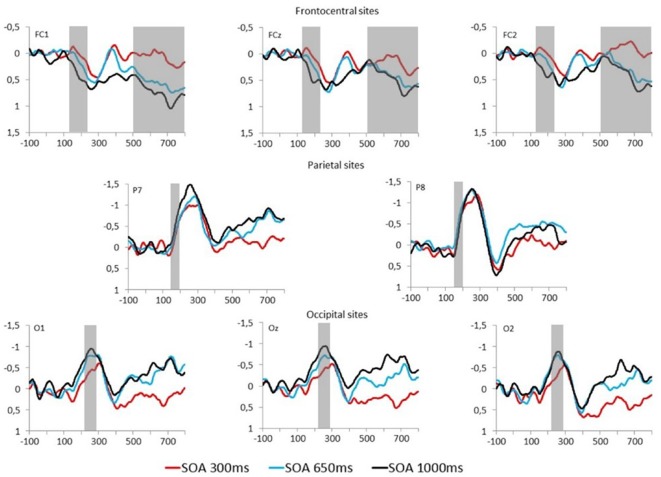
Fearful–Neutral difference waveforms at frontocentral (top), parietal (middle) and occipital (bottom) electrodes are displayed for each SOA condition (red = 300 ms, blue = 650 ms, and black = 1,000 ms). Time windows for studied event-related potentials (ERPs) are indicated by the gray overlay.

**Figure 5:**
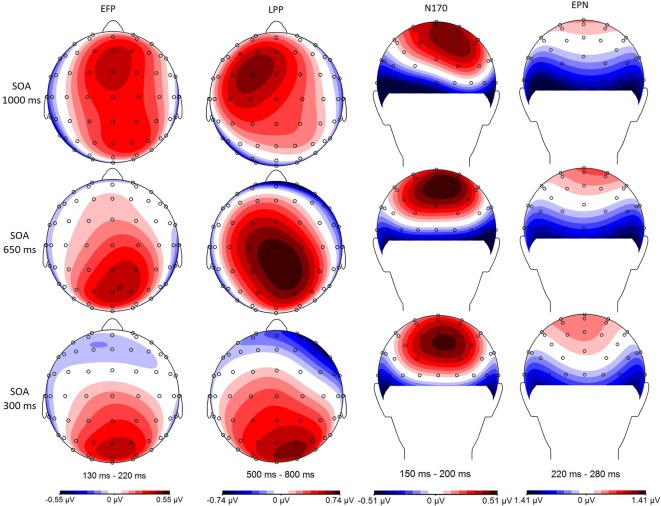
Minimal central overlap (SOA = 1,000 ms; top), and maximal overlap (SOA = 300 ms; bottom) scalp distributions are illustrated for the Fearful–Neutral difference in each of the studied time windows: 130–220 ms early frontal positivity (EFP), 150–200 ms (N170), 220–280 ms early posterior negativity (EPN), and 500–800 ms late positive potential (LPP).

**Figure 6:**
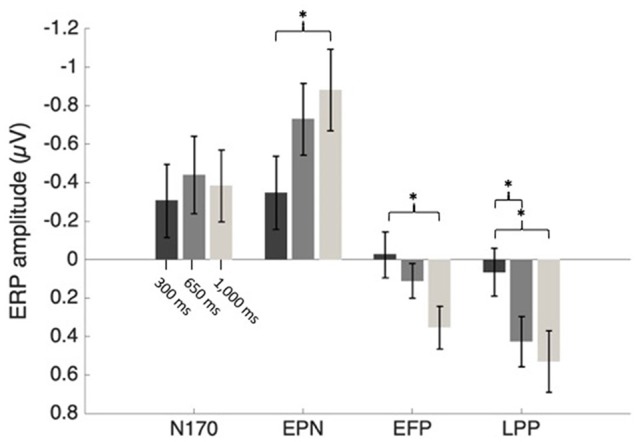
Mean ERP amplitudes as an effect of SOA condition. Error bars represent the standard error. *p < 0.05.

Fearful and neutral face waveforms are shown in Figure 2 [for frontocentral sites (EFP, LPP)], and Figure 3 [for parietal (N170) and occipital sites (EPN)], as a function of electrode and SOA. The corresponding Fearful–Neutral difference waveforms are presented in Figure 4. Scalp distributions are illustrated in Figure 5. Finally, mean amplitude of each ERP as a function of SOA is displayed in Figure 6.

#### N170

N170 mean amplitude was extracted from Fearful–Neutral difference waveform at P7 and P8 electrode sites (shown in Figure 4, middle), from 150 to 200 ms. There was no main effect of SOA or electrode, and also no SOA X Electrode interaction effect, all *F*s < 2.50. A one-sample *t*-test on the N170 difference waveform of combined electrodes and SOA conditions revealed that there was a significant emotional modulation of this ERP (*M* = −0.39 μV, *SD* = 0.55), *t*_(26)_ = −3.72, *p* = 0.001, *d* = 0.71.

#### Early Posterior Negativity

EPN mean amplitude was extracted from Fearful–Neutral difference waveform at O1, Oz and O2 electrode sites (shown in Figure 4, bottom) from 220 to 280 ms post stimulus. There was a main effect of SOA on EPN amplitude, *F*_(2,52)_ = 4.74, *p* = 0.01, $\eta_{\text{p}}^{2}$ = 0.15. One sample *t*-tests on combined electrodes revealed that the EPN significantly differed from 0 in the 1,000 ms (*M* = −0.88 μV, *SD* = 1.1, *t*_(26)_ = −4.16, *p* < 0.001, *d* = 0.8), and 650 ms (*M* = −0.73 μV, *SD* = 0.97, *t*_(26)_ = −3.91, *p* = 0.001, *d* = 0.75) SOA conditions; and marginally differed from 0 in the 300 ms SOA condition (*M* = −0.35 μV, *SD* = 0.99, *t*_(26)_ = −1.83, *p* = 0.08, *d* = 0.35). However, despite the tendency for an emotion response of the EPN in the maximal central overlap condition (SOA = 300 ms), there was an increase of this response as central overlap decreased, which was confirmed by a significant linear trend (*F*_(1,26)_ = 8.55, *p* < 0.01, $\eta_{\text{p}}^{2}$ = 0.25); and indeed, paired comparisons revealed a significant difference between EPN amplitudes in the 1,000 ms and 300 ms SOA conditions, *p* < 0.05 (Figure 4, left). There was no Electrode main effect, or SOA X Electrode interaction effect, both *F*s < 1.

#### Early Frontal Positivity

EFP mean amplitude was extracted from Fearful–Neutral difference waveforms at FC1, FCz and FC2 electrode sites (shown in Figure 4, top) from 130 to 220 ms post stimulus. There was a main effect of SOA on EFP amplitude, *F*_(2,52)_ = 4.07, *p* = 0.03, $\eta_{\text{p}}^{2}$ = 0.14. One-sample *t*-tests on combined electrodes revealed that the EFP emotion effect was significant in the 1,000 ms SOA condition (*M* = 0.35 μV, *SD* = 0.58, *t*_(26)_ = 3.17, *p* = 0.004, *d* = 0.6), but not in the 650 ms (*M* = 0.11 μV, *SD* = 0.47, *t*_(26)_ = 1.22, *p* = 0.23, *d* = 0.23) and 300 ms SOA conditions (*M* = −0.03 μV, *SD* = 0.62, *t*_(26)_ = −0.22, *p* = 0.83, *d* = 0.05). There was, once again, an increase of amplitude as central overlap decreased, which was confirmed by a significant linear trend (*F*_(1,26)_ = 6.59, *p* < 0.05, $\eta_{\text{p}}^{2}$ = 0.2); and indeed, paired comparisons revealed a significant difference between EFP amplitudes in the 1,000 ms and 300 ms SOA conditions, *p* < 0.05 (Figure 4, middle). There was no Electrode main effect or SOA × Electrode interaction, both *F*s < 1.

#### Late Positive Potential

LPP mean amplitude was extracted from Fearful–Neutral difference waveforms at FC1, FCz and FC2 electrode sites (shown in Figure 4, top), from 500 to 800 ms post stimulus. There was a main effect of SOA on LPP amplitude, *F*_(2,52)_ = 5.06, *p* = 0.01, $\eta_{\text{p}}^{2}$ = 0.16. One-sample *t*-tests on combined electrodes revealed that the LPP emotion effect was significant in the 1,000 ms (*M* = 0.53 μV, *SD* = 0.84, *t*_(26)_ = 3.28, *p* = 0.003, *d* = 0.63) and 650 ms (*M* = 0.43 μV, *SD* = 0.67, *t*_(26)_ = 3.29, *p* = 0.003, *d* = 0.64) SOA conditions, but not in the 300 ms SOA condition (*M* = 0.07 μV, *SD* = 0.64, *t*_(26)_ = 0.53, *p* = 0.60, *d* = 0.11). Once more, there was an increase of amplitude as central overlap decreased, which was confirmed by a significant linear trend (*F*_(1,26)_ = 7.72, *p* < 0.05, $\eta_{\text{p}}^{2}$ = 0.23); and paired comparisons revealed a significant difference between LPP amplitudes in the 1,000 ms and 300 ms SOA conditions, and also between 650 ms and 300 ms, both *p*s < 0.05 (Figure 4, right). There also was a significant main effect of electrode, *F*_(2,52)_ = 6.89, *p* = 0.002, $\eta_{\text{p}}^{2}$ = 0.21, and paired comparisons revealed that FC1 amplitude was larger, compared to FC2 amplitude, *p* < 0.01. The SOA x Electrode interaction did not reach significance, *F* < 1.

## Discussion

Given the social and evolutionary importance for the ability to nonverbally express emotions through facial actions (Ekman and Friesen, 1975), many researchers have assumed that the ability to recognize some of these emotions must be automatic (e.g., Palermo and Rhodes, 2007). Though one study is hardly enough to contradict years of empirical evidence, our results from three different ERPs directly linked to a specific facial emotion processing stages make a significant contribution to a growing body of literature inconsistent with this account, suggesting that this model should at the very least be tempered.

To this date, most studies that have investigated the question relied on visual-spatial attention paradigms; and despite initial evidence for automatic facial emotion processing (Vuilleumier et al., 2001), paradigms in which the main task was difficult enough managed to hinder processing of task-irrelevant expressive faces (e.g., Pessoa et al., 2002, 2005). Though this latter finding pointed at a potential role for central attention, few studies had so far addressed the issue from the standpoint of the PRP. Unsurprisingly, our behavioral data indicated a strong PRP effect on facial expression processing, similar to previous work (Tomasik et al., 2009; Shaw et al., 2011; Duncan et al., 2019; but see however, for contradicting findings pertaining to the analogous backward correspondence effect, Allen et al., 2017). Indeed, RT to T_2_ increased by almost 260 ms when the central overlap was largest, relative to when it was smallest, suggesting that central resources were in fact direct toward T_1_ as T_2_ was presented.

Interestingly, across all conditions, approximately 25 ms faster RTs were observed for fearful (vs. neutral) faces. This could be taken to indicate automatic fear detection. However, caution is warranted: Indeed, there was a small expression effect on T_2_ accuracy, reflecting greater detection accuracy for neutral (vs. fearful) expressions in the minimal overlap condition (SOA 1,000 ms). Thus, a slight speed-accuracy tradeoff could have been at play, rather than faster detection of fearful expressions *per se*. In addition, the robust finding of faster fear detection is somewhat at odds with findings from classic emotion categorization tasks, whereby fear is typically the expression for which response latency is slowest, proportion of errors is largest, and resilience to input noise is smallest (e.g., Duncan et al., 2017, 2019).

Thus, to get a better picture of the dynamics between perceptual processes and central attention, we also included ERP measures to our paradigm. The fact that the only previous comparable study relied on the N2pc (Shaw et al., 2011) makes drawing clear conclusions regarding perceptual processes difficult, as this ERP is at best an indirect measure of visual perceptual processing that instead indexes visual-spatial attention allocation (Luck and Hillyard, 1994; Eimer, 1996; Brisson et al., 2007). We, therefore, opted to focus on three emotion-specific ERPs, namely the EPN, EFP, and LPP, and also focus on a face-specific ERP known to exhibit emotion-sensitivity (N170). Importantly, each of our selected ERPs covered a distinct stage of facial emotion processing: face detection (N170), perceptual encoding of facial emotion (EPN), rapid emotion detection (EFP), and emotional content evaluation (LPP). These also encompassed a varied time-course [130–220 ms (EFP), 150–200 ms (N170), 220–280 ms (EPN), and 500–800 ms (LPP) post-stimulus], and a distinct topography [posterior (N170, EPN), frontal (EFP, LPP)].

ERP amplitudes, computed as the difference between fearful and neutral faces, were measured at three levels of central overlap as participants completed a fearful facial expression detection task that was embedded within a PRP paradigm. In contrast to previous PRP studies (eg., Shaw et al., 2011), our prioritized auditory task was more difficult as indicated by overall slower RT compared to such studies. Furthermore, the three SOAs were chosen so that the interval between response to T_1_ and T_2_ onset would be too short to allow a dynamic shift in task preparation from the first to the second task. T_2_ onset thus occurred before mean RT_1_ in the two shortest SOA conditions (300, and 650 ms), and only 27 ms after mean RT_1_ in the longest SOA condition (1,000 ms).

In our minimal central overlap condition (SOA = 1,000 ms), all ERP amplitudes differed from zero, confirming their sensitivity to emotional faces (for review, see also Eimer and Holmes, 2007; Vuilleumier and Pourtois, 2007; Hajcak et al., 2011). In our maximal central overlap condition (SOA = 300 ms), on the other hand, EFP and LPP amplitudes were statistically null, indicating an absence of emotion-sensitivity; and EPN amplitude was significantly reduced—being only marginally different from zero—indicating a reduction of emotion-sensitivity. Interestingly, all these effects followed a linear trend, indicating that interference is proportional to the degree of central overlap.

These results present both similarities and differences with the study of Holmes et al. (2009). Indeed, these authors observed a similar reduction of early and late frontocentral emotional effects in a high cognitive load (2-back) condition. Interestingly, this was only true of low, but not high trait anxiety individuals. Thus, it appears that idiosyncratic factors, such as motivational saliency of emotional content, may influence the extent to which information processing stages are resilient to central interference.

Contrary to our study however, Holmes and colleagues found no evidence of EPN attenuation under high cognitive load (see also Müller-Bardorff et al., 2016). They suggested that under circumstances in which emotional stimuli fall within focal attention, visual sensory signals could be enhanced independently from the availability of information processing resources. Even though faces were both task-relevant and presented in central vision in our study, we still observed EPN amplitude attenuation as a result of central resource scarcity. Several methodological aspects could account for this discrepancy, making it difficult to draw conclusions with any degree of certainty. However, our study does make two notable improvements in this regard: first, it excludes the confounding effect of task preparation, and second, it eliminates the influence of low-level image variance on ERP difference waveforms.

As to what process specifically could be altered by central resource scarcity at the EPN level, one possibility pertains to this EPN indexing emotional facial cue extraction (Schupp et al., 2004; Schönwald and Müller, 2014; Bekhtereva et al., 2015). Most relevant to this effect is a recent study that, combining a reverse correlation psychophysical method with the PRP paradigm, has revealed a selective left eye processing deficit under central load that generalizes across five of the six basic facial emotions—including fearful expressions (Duncan et al., 2019). The present finding of decreased EPN amplitude under central load could thus reflect such a selective emotional facial cue processing deficit. Future research should, therefore, investigate this possibility.

In contrast to the EPN, EFP, and LPP, emotional modulation of N170, on the other hand, appeared insensitive to central resource scarcity. This is especially interesting, considering that the effect cannot be attributed to low-level visual differences. It may therefore very well be the case that it was emotional facial content *per se* that drove the difference in amplitude between neutral and fearful expressions. It should, however, be noted that, though stimulus-specific factors like color and cropping do not appear to be driving the emotional N170 modulation, other factors might and those are still not well understood (Hinojosa et al., 2015).

There therefore remains alternative explanations to N170 amplitude modulation. For instance, rather than emotional content *per se*, the effect could be linked to saliency of the eye region. Indeed, within faces, the N170 is especially responsive to information from the eyes (e.g., Rousselet et al., 2014), which are particularly relevant for facial expressions of fear. Indeed, fear recognition is uniquely dependent on this feature, and failure to attend to (Adolphs et al., 2005)—or inability to use (Fiset et al., 2017)—eye information will cause a marked deficit in recognition. Fearful eyes are especially salient, so much so that some suggest that this is in fact what drives the amygdala response to fearful faces (Whalen et al., 2004). Thus, it would be of great theoretical importance to disambiguate N170 eye-sensitivity, and N170 emotion-sensitivity.

A potential limitation of the present work pertains to the fact that scalp EEG recordings cannot reliably capture subcortical activity, such as that induced by emotion-specific processing in the amygdala. Previous findings suggested that threat-related stimuli are processed within multiple neural stages and pathways, some dependent on the amygdala, and others not (Rotshtein et al., 2010). Since the present results are restricted to cortical electrical signals, it could be argued that the processes concerned here only occur after subcortical pre-attentive (i.e., automatic) emotion detection processes (e.g., Eimer and Holmes, 2007; Palermo and Rhodes, 2007). However, results from intracranial electrophysiological recordings indicate that without priming or task preparation, significant processing of emotional information in the amygdala only appears around 140 ms post-stimulus (Willenbockel et al., 2012), similar to EFP onset time. Moreover, a recent functional neuroimaging study has shown that facial emotion processing in the amygdala is disrupted by a high cognitive load on executive functions (Sebastian et al., 2017), joining numerous others indicating that emotion processing in the amygdala is not entirely automatic and may at least require some attentional resources (Pessoa et al., 2002, 2005; Bishop et al., 2007; Mitchell et al., 2007; Alpers et al., 2009; Kellermann et al., 2011). Thus, emotion processing occurring in the amygdala might have also been affected by our experimental paradigm—though future research will be needed to make an assertion on this issue.

In conclusion, the present results provide for a better understanding of the cortical processing of facial expressions of emotions. They make it clear that crucial parts of this chain of events—namely, perceptual encoding, emotion detection, and emotional content evaluation, as indexed by the EFP, EPN, and LPP, respectively—all depend to an extent on central resource availability. Processing indexed by the N170, on the other hand, does not appear to be sensitive to central resource scarcity; however, we cannot definitively conclude that this processing is emotion-specific. Thus, even if automatic processing of potential social threats would provide a clear advantage from an evolutionary standpoint, some aspects of facial emotion processing are clearly not fully automatic.

## Data Availability Statement

The datasets generated for this study are available on request to the corresponding author.

## Ethics Statement

All experiments involving human participants were reviewed and approved by the Comité d’éthique de la Recherche sur les êtres Humains, Université du Québec à Trois-Rivières. Participants provided their written informed consent to participate in this study.

## Author Contributions

AR, JD, DF, and BB conceived the study. AR and JD collected data. AR, JD, and BB performed statistical analyses. All authors contributed to data interpretation, final revision, read, and approved the submitted manuscript. AR wrote the first draft of the manuscript. AR and JD co-wrote subsequent versions. JD, DF, and BB critically revised the manuscript.

## Conflict of Interest

The authors declare that the research was conducted in the absence of any commercial or financial relationships that could be construed as a potential conflict of interest.
